# Analysis of the Role of the SRC Tyrosine Kinase and Podoplanin in the Process of Entosis

**DOI:** 10.3390/cancers17193173

**Published:** 2025-09-29

**Authors:** Agata M. Gawel, Marlena Godlewska, Lukasz P. Biały, Izabela Mlynarczuk-Bialy

**Affiliations:** 1 Department of Histology and Embryology, Faculty of Medicine, Medical University of Warsaw, 02-004 Warsaw, Poland; lbialy@wum.edu.pl; 2 Department of Clinical Physiology, Centre of Postgraduate Medical Education, 01-813 Warsaw, Poland; 3 Department of Cellular Biology and Immunology, Centre of Postgraduate Medical Education, 01-813 Warsaw, Poland; marlena.godlewska@cmkp.edu.pl

**Keywords:** entosis, breast cancer, pancreatic cancer, SRC tyrosine kinase, podoplanin

## Abstract

**Simple Summary:**

Diagnosis and treatment of cancer constitutes a significant social and clinical problem due to the limited number of effective therapeutic strategies, resulting, among other reasons, from the still poorly understood biology of tumors. It is considered that one of the processes by which cancer cells might survive the host immune response and chemotherapies is their ability to form “entosis” (cell-in-cell structures), the main features of which are the presence of one cell inside another and a crescent-shaped nucleus surrounding the inner cell. Entotic figures are observed in selected cell lines/tissues derived from aggressive tumors, and a high number of entoses are associated with a more advanced tumor stage and worse prognosis. Therefore, our goal was to gain new knowledge concerning entosis by defining new molecular drivers of this process. We believe that, in the end, the identified pro-entotic factors may be clinically beneficial as specific biomarkers indicating the biological status/advancement of the tumor.

**Abstract:**

Background: Over the last years, the phenomenon of entosis, a form of cell-in-cell structure, has been highlighted in various tumors, including poorly treatable breast or pancreatic cancers. Nevertheless, not only the biological properties, but also the molecular drivers of entosis remain unclear. Here, we evaluated SRC tyrosine kinase, a key proto-oncogene, and podoplanin (PDPN), a membrane glycoprotein, as potential regulators of entotic cell formation. Methods: In the study, two entosis-competent cell lines, BxPC-3 and MFC-7, originating from pancreatic and breast cancers, respectively, were used. *SRC* or *PDPN* genes were silenced using dedicated siRNA and the frequency of entotic structure formation was assessed using fluorescent staining and confocal imaging. Results: It was found that BxPC-3 cells deficient in *PDPN* are more prone to form entotic structures and that over 90% of all entotic figures formed by mixed PDPN^+^ and PDPN^-^ BxPC-3 cells involved *PDPN*-silenced cells. The SRC data supports this observation, as the suppressed entotic formation ability presented by *SRC*-deficient cells was linked with increased expression of *PDPN*. Even though the observed effects were mainly limited to BxPC-3 cells, as *PDPN* expression in MCF-7 cells is restricted, overall, the obtained data suggest a strong anti-entotic function of PDPN. Additionally, the performed Western blotting indicated the activation of ezrin-radixin-moesin (ERM) proteins in *PDPN*-deficient cells. Conclusions: Taken together, these data suggest that the negatively controlled PDPN-ERM axis may act as a molecular factor controlling the development of entotic structures and cells with naturally low *PDPN* expression may be more liable to form entoses.

## 1. Introduction

Despite increased reporting of novel anti-tumor strategies that have promised to prolong the survival of cancer patients, the available primary anticancer therapies offer only limited advantages, especially for curing poor-prognosis tumors characterized by high aggressiveness. It is considered that, among others, one of the mechanisms by which cancer cells may escape anticancer therapies is by either entering or being engulfed by a neighboring cell, which results in formation of a so-called entotic structure [1,2]. 

To identify an entotic figure, Mackay et al. [3] proposed several features which must be present to recognize this subtype of cell-in-cell (CIC) structure, including the following: visualization of the nucleus and cytoplasm of both the inner and engulfing cell, a half-moon (crescent)-shaped nucleus of the outer cell, and the presence of a vacuole encompassing the inner (engulfed) cell [4,5,6]. Entotic cells have been reported in pancreatic cancer, triple-negative breast cancer, gastric cancer, or head and neck squamous cell carcinoma [7,8,9]. Studies, including our own, have shown that this phenomenon may be linked to tumor progression, as higher rates of entoses were observed in metastases of breast cancer [4]. It has been reported that the frequency of entoses is associated with a high Ki-67 index and lymph node metastasis in patients with HER2-positive mammary breast cancer [5]. Similarly, Wen et al. [10] reported that the rate of entotic structures was higher in patients with castration-resistant human prostate cancer than in those with benign prostate hyperplasia or androgen-dependent prostate cancer. Also, it is considered that by “hiding” within a neighboring cell, entotic figures are capable of escaping therapeutic drugs; therefore, entosis may be of special importance in the resistance of tumor cells to administered drugs, such as the tyrosine kinase inhibitor nintedanib [11]. The fate of the entotic cells may either be the escape and survival of both, engulfed and engulfing cells, but also death via lysosomal degradation is frequent [2,12]. Nevertheless, the biological role, as well as the detailed mechanistic processes and factors involved in entosis, still remain unclear.

Here, we aim to expand the knowledge regarding the molecular factors controlling entosis by investigating the role of two pro-carcinogenic markers, i.e., podoplanin (PDPN) and SRC tyrosine kinase (SRC), as pro-entotic factors. We were particularly interested in evaluating the direct consequences of depletion of *PDPN* or *SRC* on the frequency of entotic formation, as well as the susceptibility of the studied cells to act as engulfed or engulfing cells. 

PDPN (encoded by the *PDPN* gene) is a membrane glycoprotein composed of three domains: ~130 amino acid N-terminal extracellular, transmembrane, and ~10 amino acid intracellular domain [13]. The intracellular tail of PDPN interacts with ezrin-radixin-moesin (ERM) proteins, protein kinase A (PKA), and cyclin-dependent kinase 5 (CDK5). PDPN and ERM family members, which trigger RhoA activation, have been proposed as a potential mechanism of the promotion of cellular epithelial-to-mesenchymal transition (EMT) and metastasis [14]. Also, PDPN expression has been directly linked to various factors of signaling pathways, including RhoA and RAC1 GTPases, and their regulators. It was implied that high PDPN expression leads to the overactivation of RhoA [15], a central and crucial mechanotransduction regulator within focal adhesions [16]; its activation involves extensive crosstalk between integrins, SRC family kinases, and between individual Rho GTPases themselves [17]. PDPN is involved in cancer cell migration, and elevated levels of this protein have been observed in some malignant tumors, such as squamous cell carcinomas or gliomas, as well as cancer-associated fibroblasts in breast and pancreatic tumors [13]. The migratory potential of PDPN has been associated with the genetic composition of cancer, including absence or presence of certain mutations like *RET*/PTC1 or *BRAF*V600E [15]. 

The SRC protein-tyrosine kinase is a broadly studied proto-oncogene engaged in numerous processes involved in tumor formation. SRC is upregulated in various tumors, including breast or prostate cancer, and its overexpression is linked with worse patient survival [18,19]. SRC is recognized as a key regulator of motility [20]. It stimulates the migratory potential and adhesion of cancer cells by affecting the signal transduction network of the mitogen-activated protein kinase (*MAPK*) or PI3K/Akt cascades [19,21]. Some data suggests that indirect inhibition of SRC activity may prevent an increase in entotic numbers [22]. 

Importantly, SRC has been reported to induce the expression of *PDPN* via the phosphorylation of the adaptor Crk-associated substrate (Cas) protein and it was indicated that *PDPN* acts downstream of SRC and Cas to promote cell migration [23]. In recent studies, Retzenbach et al. [24] suggested that both SRC and PDPN are required to promote transformed cell growth and motility in complementary, but parallel pathways. Therefore, here, the effects of *SRC* and *PDPN* expression in entosis were examined jointly. 

## 2. Materials and Methods

### 2.1. Cell Lines

The MCF-7 cells were purchased from the Leibniz Institute DSMZ-German Collection of Microorganisms and Cell Cultures GmbH (Number: ACC 115; Braunschweig, Germany) and grown in Dulbecco’s Modified Eagle Medium (DMEM; Corning, Inc., Corning, New York, NY, USA; Sigma-Aldrich; St. Louis, MO, USA) supplemented with 10% fetal bovine serum (FBS; HyClone, Cytiva; Marlborough, MA, USA). The BxPC-3 cell line was acquired from the American Type Culture Collection (ATCC; Number: CFL-1687; Manassas, VA, USA) and cultured in Roswell Park Memorial Institute medium 1640 (RPMI-1640; Corning, Inc.) supplemented with 10% FBS (Cytiva). Both cell lines were grown at 37 °C in a humidified incubator with 5% CO_2_.

### 2.2. Gene Silencing Using Small Interfering RNA (siRNA)

The expression of the *SRC* and *PDPN* genes was silenced using validated small interfering RNA (siRNA; 30 nM): siSRC (siRNA ID: s13414; validated Silencer Select; or/and siRNA ID: s13413; Silencer Select; ThermoFisher Scientific; Waltham, MA, USA) and siPDPN used in previous studies (e.g., [15]) (siRNA ID: s20886 or/and s225443; Silencer Select; ThermoFisher Scientific), respectively. MCF-7 and BxPC-3 cells transfected with MISSION siRNA Universal Negative Control #1 (siNEG; Sigma-Aldrich; St. Louis, MO, USA) served as controls. For gene knockdown, the respective siRNA was diluted in Opti-MEM I Reduced Serum Medium (Gibco; ThermoFisher Scientific) and combined with Lipofectamine 2000 Reagent (Invitrogen; ThermoFisher Scientific) diluted in Opti-MEM in a 1:1 ratio, as already described [25]. Transfection efficiency was confirmed using the RT-qPCR and Western blot techniques.

### 2.3. Analysis of Entotic Figures Using Confocal Microscopy

After gene silencing, the MCF-7 and BxPC-3 cells transfected with siNEG, siSRC, or siPDPN were independently seeded onto µ-Slide 8 Well chambered coverslips (Ibidi; Grafelfing, Germany). After 24 h of incubation at 37 °C in a CO_2_ incubator, the cells were washed in phosphate-buffered saline (PBS) supplemented with 2% bovine serum albumin (BSA; Sigma-Aldrich) for 30 min, stained using phalloidin-FITC (fluorescein isothiocyanate; 2 µg/mL in PBS; Sigma-Aldrich) for 40 min, followed by washing with PBS. Next, the cells were stained with Hoechst 33342 dye (NucBlue Live ReadyProbes Reagent; Sigma-Aldrich) for 2 min and washed using distilled water. All steps were performed at room temperature, unless otherwise stated. The slides were imaged using a Zeiss LSM800 confocal unit equipped with a plan-apochromatic 63x/1.4 oil DIC M27 lens (Carl Zeiss AG; Oberkochen, Germany). Entoses were positively identified using Mackay’s criteria [3]. The percentage of entotic figures for each analyzed microscopic field was calculated according to the equation: Entotic frequency [%] = [(Number of entoses)/(Total number of cells)] × 100. Next, an average of all entotic frequencies for both cell lines and each condition (siNEG, siSRC, and siPDPN) was calculated independently.

### 2.4. Co-Culture Experiment for the Evaluation of Entotic Structures

To further assess the nature of the formed entotic figures, a co-culture experiment with cell staining using cell-tracker dyes was performed. At 24 h post-transfection, the MCF-7 and BxPC-3 cells treated with siNEG were stained using CellTracker Red CMTPX (10 µM; Invitrogen; ThermoFisher Scientific) for 60 min, while the cells transfected with siSRC and siPDPN were each stained using CellTracker Green CMFDA (10 µM; Invitrogen; ThermoFisher Scientific) for 40 min. Next, the control and siPDPN-treated cells were mixed and seeded on microscope circular coverslips placed in a 24-well plate. The same procedure was conducted for the *SRC*-depleted cells (control and siSRC-treated cells were pooled together). After 24 h of co-culture, the cells were washed using Dulbecco’s PBS without calcium and magnesium (D-PBS; Corning, Inc.), fixed in 4% paraformaldehyde (PFA; ThermoFisher Scientific) for 10 min, washed twice with PBS, followed by staining using Hoechst 33342 (Sigma-Aldrich) for 2 min, and finally washed using deionised water. Subsequently, the slides were fixed on frosted glass microscope slides using Fluorescence Mounting Medium (Dako; Carpinteria, CA, USA) and imaged using the Zeiss LSM800 confocal unit (Carl Zeiss AG). Entotic frequencies were calculated for each cell line and condition, according to the equation presented above. 

### 2.5. RNA Extraction, Reverse Transcription, and RT-qPCR

To confirm the effective down-regulation of the genes encoding *SRC* and *PDPN*, RNA extraction, cDNA synthesis, and RT-qPCR were performed, as already described [26]. Briefly, forty-eight hours after transfection, total RNA was extracted using RL buffer (EURx; Gdansk, Poland) with added beta-mercaptoethanol (Sigma-Aldrich) and then purified using the Universal RNA Purification Kit (EURx). RNA purity was assessed on the NanoDrop One Spectrophotometer (ThermoFisher Scientific). cDNA synthesis and RT-qPCR were conducted on the CFX96 Detection System (Bio-Rad; Hercules, CA, USA) using a High-Capacity cDNA Reverse Transcription Kit with RNase Inhibitor (Applied Biosystems; ThermoFisher Scientific) and 5x HOT FIREPol EvaGreen qPCR Mix Plus (Solis BioDyne; Tartu, Estonia), respectively, following the manufacturer’s instructions. The sequences of the used RT-qPCR oligonucleotide primers are listed below:

*18S rRNA*: 

Forward: 5′-CCAGTAAGTGCGGGTCATAAG; 

Reverse: 5′-CCATCCAATCGGTAGTAGCG;

*SRC*: 

Forward: 5′-CTGCTTTGGCGAGGTGTGGATG; 

Reverse: 5′-CCACAGCATACAACTGCACCAG;

*PDPN*: 

Forward: 5′-CGAAGATGATGTGGTGACTC;

Reverse: 5′-CGATGCGAATGCCTGTTAC. 

### 2.6. Western Blot Analysis

Effective knockdown of *SRC* and *PDPN* was additionally confirmed using the Western blot technique, as previously described [15]. Briefly, cells were seeded and grown on 6-well plates. At 72 h post-silencing, total protein was extracted using a cocktail composed of the following: the radioimmunoprecipitation assay (RIPA) buffer (ThermoFisher Scientific), Pierce Phosphatase Inhibitor Cocktail (ThermoFisher Scientific), complete Protease Inhibitor Cocktail (Roche; Mannheim, Germany), and Viscolase (a recombinant nuclease; A&A Biotechnology; Gdansk, Poland). Total protein concentration was determined using the BCA Protein Assay Kit (ThermoFisher Scientific) and Synergy 2 Multi-Mode Microplate Reader (BioTek Instruments, Inc.; Winooski, VT, USA). Then, standard Western blot analyses were performed using primary, followed by incubation with secondary, antibodies. All the used antibodies, including incubation conditions, are summarized in Table 1. Signals from reactive bands were visualized with the SuperSignal West Dura Extended Duration Substrate (ThermoFisher Scientific) and captured using the Mini HD9 acquisition system (Uvitec Ltd.; Cambridge, UK). Densitometry (using the National Institute of Health’s ImageJ 1.54g software, [27]) was used to quantify protein bands on blots by measuring the integrated density (intensity) of pixels within selected regions.

### 2.7. Immunocytochemistry Analysis

To determine the cellular level and localization of targeted proteins, the MCF-7 and BxPC-3 cells treated with siRNA (siNEG, siSRC, or siPDPN; as described above) were seeded on microscope circular coverslips placed in a 24-well plate. After 24 h of culture, the cells were washed using D-PBS (Corning, Inc.), fixed in 4% PFA (ThermoFisher Scientific) for 10 min, and washed three times using PBS. Next, the cells were permeabilized for 5 min using 0.5% TBST (Tris-buffered saline with 0.5% Tween 20; Sigma-Aldrich) and then washed with PBS. This was followed by a 1 h incubation with a blocking buffer consisting of 3% BSA (Sigma-Aldrich) and 3% normal goat serum (Sigma-Aldrich) in a PBS. After blocking, the cells were probed overnight at 4 ℃ with mouse monoclonal antibodies specific for total SRC (Cat No. 2110; Cell Signaling Technology, Inc.) or PDPN (Cat No. MCA2543; Bio-Rad), each diluted 1:500 in PBS. Next, the cells were washed with PBS, probed using a secondary antibody (anti-mouse IgG Fab2 Alexa Fluor 594 Conjugate; Cell Signaling Technology) diluted at a ratio of 1:10,000 in PBS for 1 h, and subsequently washed extensively in PBS. This step was followed by staining with phalloidin-FITC (2 µg/mL in PBS; Sigma-Aldrich) for 45 min and Hoechst 33342 solution (ThermoFisher Scientific) for 10 min. After staining, the cells were extensively washed with PBS and fixed on frosted glass microscope slides using mounting medium, as described in Section 2.4. All steps were performed at room temperature, unless otherwise indicated.

### 2.8. Cell Viability Assay

To evaluate the effect of gene silencing on cell viability, a trypan blue exclusion assay was performed, as described elsewhere [26]. Briefly, cells treated with the transfection mixture for 72 h were detached, centrifuged, resuspended in D-PBS (Corning, Inc.), and stained with trypan blue dye (final concentration: 0.2%; NanoEnTek, Inc.; Seoul, Korea). An automated cell counter (EVE; NanoEnTek, Inc.) was used to determine the total and viable cell count. Results are expressed as a percentage of viable cells.

### 2.9. Statistical Analysis

Data analysis was performed using Prism 7.0 Windows (GraphPad Software, Inc.; La Jolla, CA, USA). Median frequencies and standard deviation (SD), and nonparametric one-way analysis of variance (ANOVA) followed by Tukey’s posthoc were performed. Densitometry data were analyzed using a one-way ANOVA test. Statistical significance was considered at *p* < 0.05. All analyses were performed at least in triplicates.

## 3. Results

The study was performed on human-derived cancer cell lines: (i) BxPC-3, an adherent cell line exhibiting epithelial morphology, isolated from the pancreatic tissue of a 61-year-old female patient with adenocarcinoma, and (ii) MCF-7, an epithelial cell line isolated from the breast tissue of a 69-year–old female patient with metastatic adenocarcinoma. The capability to form entotic figures by both cell lines has been confirmed (these cell lines are referred to as “entosis-competent”) [8,28]. Both cell lines are recommended and widely used as a model tool to study the process of entosis. 

### 3.1. Silencing of the Expression of SRC Tyrosine Kinase and Podoplanin in Pancreatic and Breast Cancer-Derived Cells

**Figure 1:**
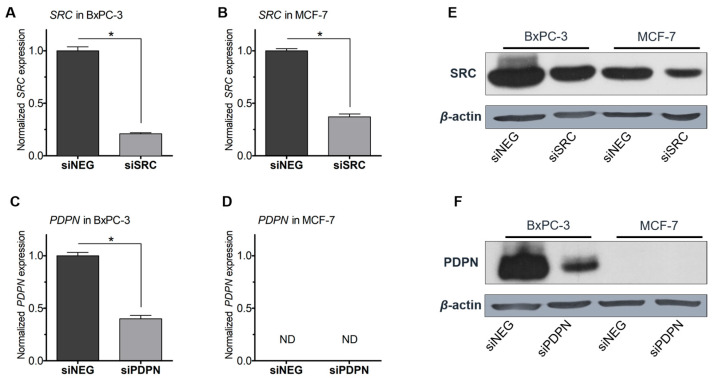
Expression of *SRC* and *PDPN* in BxPC-3 and MCF-7 cells assessed using RT-qPCR (**A**–**D**) and Western blotting (**E**,**F**). Densitometry quantifying the intensity of the bands on the blots correlated with RT-qPCR results (Figure S1). Control (siNEG)—cells transfected with negative siRNA; siSRC—cells transfected with siRNA targeting *SRC*; siPDPN—cells transfected with siRNA targeting *PDPN*; *β*-actin—loading control. * *p* < 0.05. ND—not defined; *PDPN* expression was not detected in MCF-7 cells.

To evaluate the role of SRC and PDPN in entosis, MCF-7 and BxPC-3 cells were treated with specific validated siRNA targeting SRC (siSRC) or PDPN (siPDPN) to achieve the conditions in which the yield of the studied proteins would be significantly suppressed. Cells transfected with negative siRNA (siNEG) served as controls. Efficient siRNA-mediated reduction in expression of *SRC* and *PDPN* (>3-fold) was achieved (Figure 1). Importantly, the lack of expression of *PDPN* in MCF-7 cells was observed, and this stays in accordance with previously reported data (proteinatlas.org/ENSG00000162493-PDPN/cell+line). Both *PDPN* expression and PDPN yield in MCF-7 cells were below the limit of detection of the RT-qPCR (Ct > 36) and Western blot methods, respectively.

**Figure 2:**
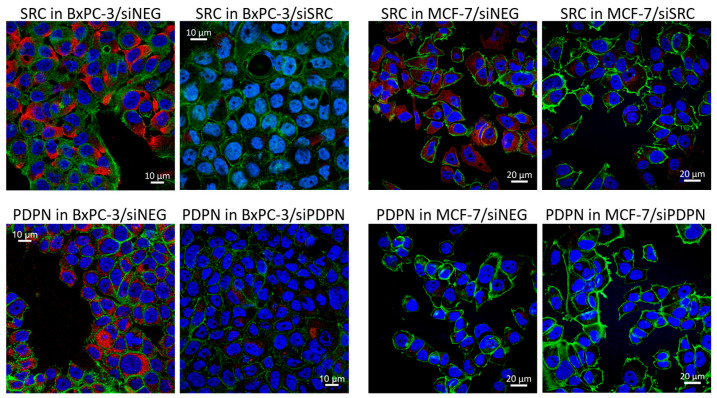
Expression of *SRC* and *PDPN* in the BxPC-3 and MCF-7 cells after transfection with siSRC or siPDPN, respectively. The red fluorescence signal shows SRC in BxPC-3 and MCF-7 (**upper row**) or PDPN in BxPC-3 (**bottom row**) cells. Green (phalloidin-FITC) and blue (Hoechst 33342) fluorescence signals mark the cytoskeleton and nuclei, respectively. siNEG—control cells; siSRC—cells transfected with siSRC; and siPDPN—cells transfected with siPDPN. FITC, fluorescein isothiocyanate.

Next, immunocytochemistry and confocal imaging were performed to monitor the level of changes in the yield and intracellular localization of SRC and PDPN in the tested pancreatic and breast cancer-derived cells after transfection with dedicated siRNA. Both the control cells (treated with siNEG) and cells transfected with siPDPN or siSRC were subsequently probed with the PDPN or SRC primary antibody, respectively, next with a secondary antibody, followed by phalloidin and Hoechst staining (Figure 2). A reduced signal of SRC and PDPN (red signal) after treatment with siSRC or siPDPN, respectively, was observed for both cell lines, confirming that the knockdown of the genes of interest was efficient. In the control and siPDPN-treated MCF-7 cells, presence of PDPN was not detected, which further supports the above data (Figure 1) and confirms lack of expression of PDPN in the MCF-7 cell line.

### 3.2. Depletion of SRC and PDPN Does Not Affect Cell Viability

**Figure 3:**
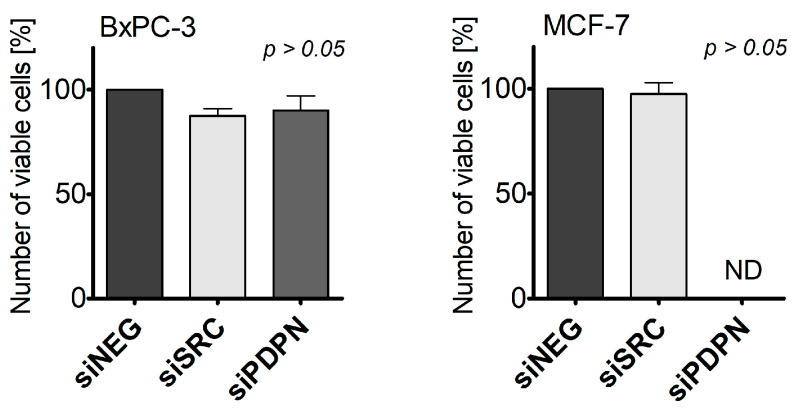
Analysis of the viability of BxPC-3 and MCF-7 cells with silenced *SRC* and *PDPN* genes using the trypan blue exclusion assay. Viability represented as change relative to control (siNEG); siSRC—cells transfected with siSRC; and siPDPN—cells transfected with siPDPN. *p* > 0.05. ND-not defined.

The viability of the cells was determined to confirm that *SRC* and *PDPN* knockdown does not result in changes in survival of the assessed cells, which might directly affect the frequency of entotic formation. As shown in Figure 3, no significant changes in survival rates for both cell lines after treatment with siRNA were observed. These data ensured that potential shifts in entotic cell formation patterns were not a consequence of altered cell death. 

### 3.3. Knockdown of SRC Reduces the Number of Entoses, While PDPN-Deficient Cells Are Entoses-Competent

**Figure 4:**
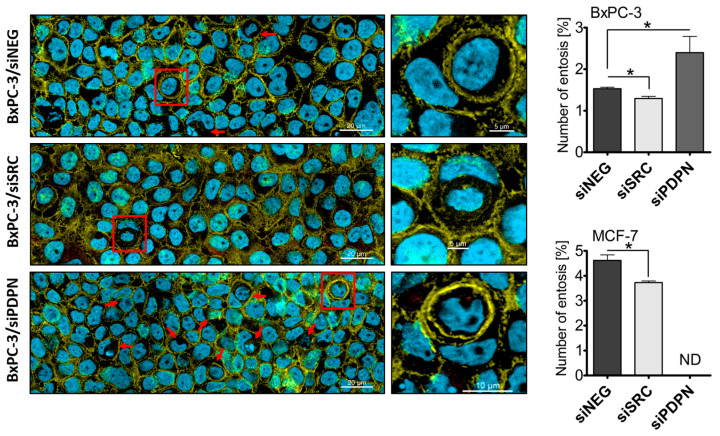
Frequency of entotic figures in *SRC*- and *PDPN*-silenced BxPC-3 and MCF-7 cells. Confocal imaging of the entotic figures in *SRC*- and *PDPN*-silenced BxPC-3 cells (left panel; marked with red arrow) and the quantification of these figures in the total cell population of *SRC*- and *PDPN*-depleted BxPC-3 and MCF-7 cells (right panel). Fluorescence signals indicate: (light green; phalloidin-FITC)—cytoskeleton; (blue; DAPI)—nuclei. Entotic cells marked with red frame were enlarged. * *p* < 0.05. ND—not defined.

To assess the number of entotic figures in the MCF-7 and BxPC-3 cells with depleted *SRC* or *PDPN*, fluorescent-based staining was performed. Cells were grown for 24 h on cover slips, fixed, and stained with phalloidin conjugated with FITC (labeling the cytoskeleton) and Hoechst 33342 (labeling the nucleus), and imaged using confocal microscopy. Each analyzed area consisted of 100 to 1000 cells. In total, for each cell line over 10,000 of each: siNEG-, siSRC-, or siPDPN-treated cells were analyzed. For the identification of entotic figures, Mackay’s criteria [3] were applied. All variants of entotic figures were considered (including standard CIC figures, entotic nested structures, or active lysosomal death of the inner entotic cell) for the calculation of total entotic frequency. Data are shown as a percentage of entotic figures in each of the tested conditions and summarized in Figure 4. For MCF-7 control (siNEG-treated) cells, ~500 entotic figures were noted in total marking the frequency of entosis around 5%. In BxPC-3 cells, as expected, the number of entotic cells was about half of that observed for the MCF-7 cell line and entotic cells consisted of up to 2% of the BxPC-3 control population. It was found that the number of entotic cells formed by both MCF-7 and BxPC-3 cells transfected with siSRC was slightly decreased. In contrast, data revealed that BxPC-3 cells with depleted *PDPN* were over twice as prone to forming CIC figures than the control cells, which indicates that PDPN may act as an inhibitor of entotic figure formation. 

### 3.4. PDPN-Deficient Cells Act as the Engulfing Cell in an Entotic Figure Formed of BxPC-3 Cells

**Figure 5:**
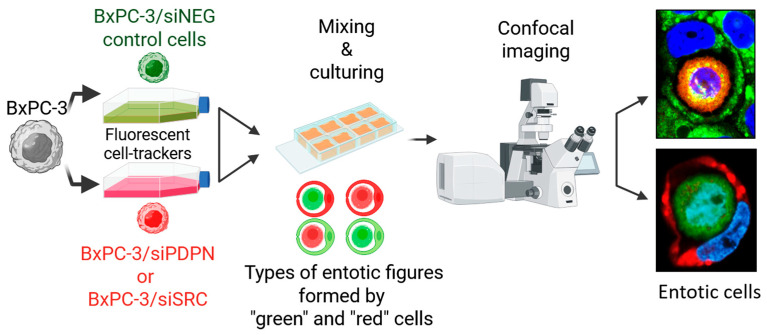
Schematic summary of the assay used to evaluate the ability of the *SRC*- or *PDPN*-deficient BxPC-3 cells to act as an inner (top image; “red in green”) or outer cell (bottom image; “green in red”) during entosis (Created in BioRender. Gawel, A. (2025) https://BioRender.com/exbq4te, accessed on 20 September 2025).

To elucidate the role of the SRC and PDPN proteins in the formation of entotic structures, as well as the strong anti-entotic properties of PDPN, we expanded our study by applying a two-color assay to analyze the nature of the CICs formed by the studied BxPC-3 cells. A co-culture experiment using two types of CellTracker dyes (green and red) was performed. As indicated in Figure 5, the control cells (siNEG) were stained using the green dye, while cells transfected with siSRC or siPDPN were stained with the red dye. Next, control cells (green) were mixed with siSRC- or siPDPN-treated cells and co-incubated. The possible types of entotic structures (evaluated using a confocal microscope) that directly reflected the outer/inner status of the cells were as follows: (i) homotypic: “red/red” or “green/green”, or (ii) heterotypic: “red/green” or “green/red” (Figure 5); where “green” were control cells and “red” were cells with the depleted gene of interest. The number of the created entotic cells of interest was calculated. This strategy allowed us to precisely determine the consequences (in the context of CIC formation) of loss of the designated protein (SRC or PDPN) and allowed us to mark if the tested cell variant was more prone to act as the “outer” or “inner” cell.

**Figure 6:**
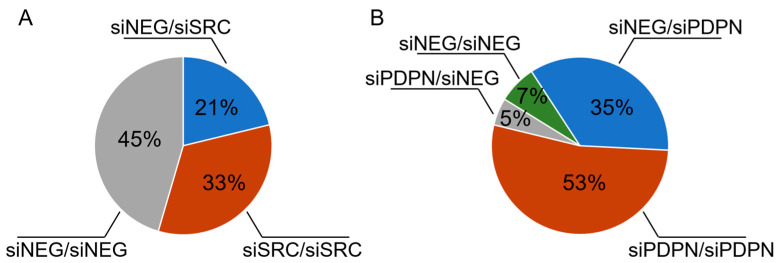
Entotic figures formed by mixed: BxPC-3 control (siNEG) and siSRC (with depleted *SRC*) cells (**A**) and BxPC-3 control (siNEG) and siPDPN (*PDPN*-deficient) (**B**) cells. Results are presented as a percentage of each type of formed entotic figures. siNEG/siNEG—control cell in control cell; siNEG/siSRC—control cell in *SRC*-depleted cell; siSRC/siSRC—*SRC*-depleted cell in *SRC*-silenced cell; siNEG/siPDPN—control cell in *PDPN*-depleted cell; siPDPN/siNEG—*PDPN*-depleted cell in control cell; siPDPN/siPDPN—*PDPN*-depleted cell in *PDPN*-silenced cell.

It was found (Figure 6) that the majority of the formed entotic figures in cells with a knockdown of *SRC* consisted of two homotypic cells, i.e., either control (siNEG) cell in another control cell (45% of the formed entoses) or an *SRC*-deficient cell within another cell with depleted *SRC* (33%). In contrast, for *PDPN*-deficient cells, entoses were more likely to consist of two *PDPN*-deficient cells (52%) or a control cell within a cell with silenced *PDPN*. Interestingly, nearly 90% of all entotic cells were found to be built of a *PDPN*-deficient cell, which acted as the outer entotic cell. This indicates that cells lacking *PDPN* are more prone to either engulf or be invaded by a neighboring cell.

### 3.5. Knockdown of SRC Increases the Expression of Podoplanin in BxPC-3 Cells

**Figure 7:**
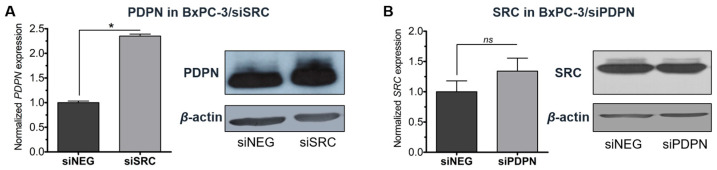
Depletion of *SRC* in BxPC-3 cells increases expression and yield of podoplanin (**A**). Reduction in *PDPN* expression does not affect SRC (**B**). siNEG—control cells; siSRC—cells with depleted *SRC*; siPDPN—cells with depleted *PDPN*. Densitometry quantification of the intensity of bands on the blots correlated with RT-qPCR results (Figure S1). Loading control: *β*-actin. * *p* < 0.05; *ns*—non-significant.

Further analysis of the BxPC-3 cells transfected with siSRC revealed that *SRC* depletion results in the activation of *PDPN* gene expression, and in consequence, to an increased yield of PDPN. Subsequently, siPDPN depletion did not affect the SRC status (Figure 7). This result stays in accordance with the data above (Figure 4), showing that *SRC* depletion suppresses the entotic process, while in contrast, *PDPN* depletion promotes formation of entotic figures. Therefore, when *PDPN* was expressed in control (siNEG) cells or in *SRC*-silenced cells, the cells were not entosis-competent. These data signify that PDPN, and not SRC tyrosine kinase, plays a direct and significant role in entotic formation.

**Figure 8:**
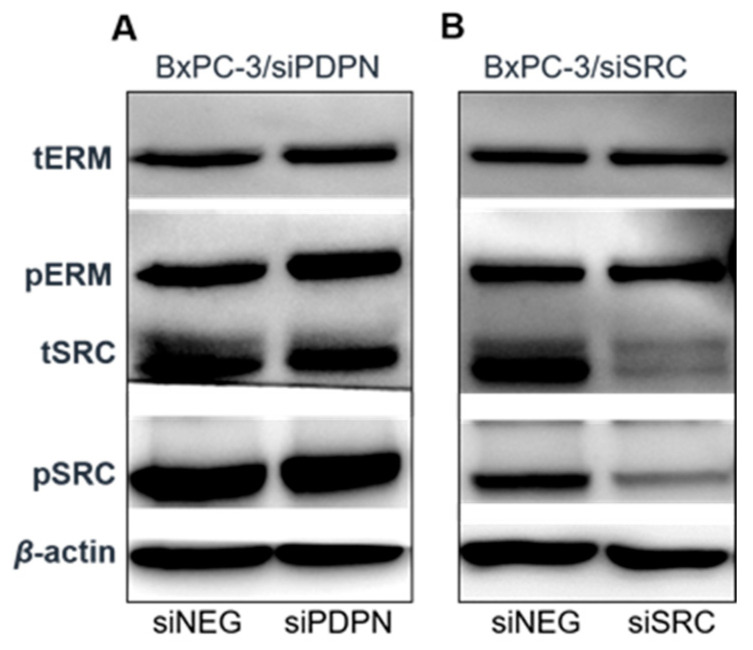
Western blot analysis of the yield of phosphorylated ezrin-radixin-moesin (ERM) proteins after depletion of *PDPN* (**A**) or *SRC* (**B**) in BxPC-3 cells. The quantification of band intensities through densitometry is presented in supplementary Figure S1. siNEG—control cells; siPDPN—cells with depleted podoplanin; pERM—phosphorylated ERM proteins; tERM—total ERM proteins; pSRC—phosphorylated SRC; tSRC—total SRC; *β*-actin—loading control.

As the obtained data imply a key regulatory function of PDPN in the process of forming entotic figures, and its role has been previously linked with the activation (phosphorylation) of ERM proteins [14], we also tested the activation status of ERM proteins in *PDPN*-depleted cells using Western blotting (Figure 8). It was revealed that loss of *PDPN* results in a modest increase in the yield of active (phosphorylated) ERM proteins (pERMs); nevertheless, the pool of total ERMs (tERMs) was unchanged. Subsequently assessed yields of SRC and phosphor-SRC were similar in the control and siPDPN samples (Figure 8).

## 4. Discussion

In the study, the role of the SRC tyrosine kinase and PDPN in entotic formation was determined. Our major finding is that PDPN may act as a crucial molecular suppressor of the process of entosis, while SRC may act as a promotor of cell-in-cell figure formation. It was revealed (Figure 4) that *PDPN*-silenced cells are twice as prone to enter each other, while *SRC* silencing led to somewhat reduced entotic figure numbers. It was concluded that the shift observed in the number of entotic cells is caused by altered survival of the cells due to depletion of the studied genes. Importantly, the role of PDPN as a potential inhibitor of entosis was further supported using a co-culture assay (Figure 6) showing that entotic cells lacking *PDPN* were more willing to develop entoses and, in total, ~90% of all the CIC structures involved at least one *PDPN*-silenced cell. Also, it was observed that PDPN was induced by inactivation of the *SRC* gene, and this increase in the level of PDPN was associated with a reduced number of entotic figures in the tested cell population. 

Overall, the data indicate that SRC might be required for the process of entosis, but we favor a model in which SRC acts as an indirect component of the machinery, affecting CIC formation via *PDPN* dysregulation (both BxPC-3 and MCF-7 cells with depleted *SRC* present a reduced capability to form entotic figures). We assumed that the data implies that PDPN is a significant, protective regulator of the process of entosis, as the entotic frequency rises in *PDPN*-depleted BxPC-3 cells. The other supportive observation showed that the reported (e.g., [9]) naturally high level of entotic cells (5–10%) correlates with a natively very low level of PDPN. Nevertheless, it is still not clear how precisely PDPN regulates entosis. 

In the process of entotic cell formation, both internal and external mechanical forces act through the cytoskeleton, influencing the mechanical properties of cells. Entosis is initiated by the calcium-dependent adhesion of two cells, with the crucial involvement of an adhesion kinase (E-cadherin), and then requires the RhoA-GTPase and Rho-kinase (ROCK)-associated actomyosin axis [8]. Additional molecular components linked with entosis include the following: the AMP-activated protein kinase (AMPK), the stress kinases c-Jun N-terminal kinase (JNK) and p38, the oncogenes Kirsten rat sarcoma 2 viral oncogene homolog (K-Ras) and cellular Myc (c-Myc), and the p53 tumor suppressor protein [2,29]. Moreover, in nonadherent culture conditions, the involvement of N-cadherin and ROCK signaling has been highlighted [30]. Importantly, Hinojosa et al. [31] also indicated the importance of up-regulation of ezrin (*EZR*) expression for the promotion of cell-in-cell invasion. We also provided evidence highlighting the potent role of the activation of ERM proteins (caused by *PDPN* depletion) in entotic cell formation. It can be considered that ERM proteins might be the end and direct factor regulating the process of CIC development. 

In general, ERM proteins are important for cell–cell and cell–matrix contacts, potentially through numerous interactions with, e.g., cadherin complexes and integrins. ERM proteins undergo activation upon binding of phosphotidylinositol 4,5-bisphosphate (PIP2) to the FERM domain and subsequent phosphorylation of the conserved threonine residues (Thr558, Thr564, Thr567 in moesin, radixin, and ezrin, respectively) [32]. It has been proposed that the phosphorylation process is mediated by several kinases, including ROCK, and this can lead to activation of different signaling pathways [33]. Therefore, it needs to be considered that not only EZR, but also radixin (RDX) and moesin (MSN) may play an important role in the formation of entotic structures, although their role still needs to be elucidated. 

## 5. Conclusions

This is the first report showing the role of the SRC tyrosine kinase and PDPN in the formation of entotic figures. The results suggest that the role of both proteins is linked and they play an important, but opposing, role in the phenomenon of entosis. SRC tyrosine kinase, which is important in cell adhesion, promotes the formation of entotic structures, while high expression of *PDPN* limits the frequency of CICs. Importantly, cells with a reduced PDPN level become entosis-competent. These observations imply a significant, anti-entotic property of PDPN. We suggest that PDPN may act as a molecular hub controlling the signal transduction necessary for the formation of entoses, and that ERM proteins might be the end molecular component regulating the frequency of formation of entotic figures. Entosis is recognized as a potential contributor to cancer progression. Still, its potential clinical value has not been established. It can be considered that the frequency of entotic cells in cancer specimens may serve as a novel, simple marker or index used as a prognostic indicator. Also, developing specific inhibitors or combinatory treatment strategies that block the molecular machinery of entosis could be another promising approach.

## Figures and Tables

**Table 1:** Primary and secondary antibodies used in the study for Western blot analysis.

Antigen	Catalog No.	Type/ Clone (Symbol)	Dilution/ Blocking Agent	Source
β-actin	3700	Mouse monoclonal (IgG2b)/ 8H10D10	1:2000/ 5% skimmed milk	Cell Signaling Technology, Inc. (Beverly, MA, USA)
pERM (T567/Ezrin, T564/Radixin, T558/Moesin)	ab76247	Rabbit monoclonal (IgG)/ EP2122Y	1:2000/ 5% BSA	Abcam (Cambridge, UK)
tERM	3142	Rabbit polyclonal	1:1000/ 5% BSA	Cell Signaling Technology, Inc.
PDPN	MCA2543	Mouse mococlonal (IgG1)/ D2-40	1:1000/ 5% skimmed milk	Bio-Rad (Hercules, CA, USA)
pSRC (Y416)	2101	Rabbit polyclonal	1:1000/ 5% BSA	Cell Signaling Technology, Inc.
tSRC	2110	Mouse mococlonal (IgG1)/ L4A1	1:1000/ 5% skimmed milk	Cell Signaling Technology, Inc.
anti-mouse secondary immunoglobulins	115-035-146	Goat polyclonal	1:10,000/ 1% skimmed milk	Jackson ImmunoResearch (West Grove, PA, USA)
anti-rabbit secondary immunoglobulins	P0448	Goat polyclonal	1:5000/ 1% skimmed milk	Dako (Carpinteria, CA, USA)

BSA, bovine serum albumin; p, phosphorylated protein; t, total protein.

## Data Availability

The data presented in this study are available in the article. Further inquiries can be directed to the corresponding author.

## Supplementary Materials

The following supporting information can be downloaded at: https://www.mdpi.com/article/10.3390/cancers17193173/s1, Figure S1. Densitometric analysis of Western blot results presented in the main manuscript: (A) Expression of total SRC and podoplanin (PDPN) in BxPC-3 and MCF-7 cells presented in Figure 1; (B) Effect of *SRC* or *PDPN* depletion in BxPC-3 cells on the yield of total SRC or podoplanin, respectively, presented in Figure 7; (C) Analysis of the yield of phosphorylated and total ERM proteins and phosphorylated and total SRC after depletion of *PDPN or SRC* in BxPC-3 cells presented in Figure 8. siNEG—control cells; siPDPN—cells with depleted *PDPN*; tSRC—total SRC; pSRC—phosphorylated SRC; tERM—total ERM (ezrin-radixin-moesin); pERM—phosphorylated ERM proteins. * *p* < 0.05; *ns*—non-significant.

## Abbreviations

The following abbreviations are used in this manuscript:

ATCC	American Type Culture Collection
BSA	Bovine serum albumin
CIC	Cell-in-cell
DAPI	4’,6-diamidino-2-phenylindole dihydrochloride
DMEM	Dulbecco’s Modified Eagle Medium
D-PBS	Dulbecco’s PBS
HRP	Horseradish peroxidase
FBS	Fetal bovine serum
FITC	Fluorescein isothiocyanate
NEG	Negative
PBS	Phosphate-buffered saline
PDPN	Podoplanin
pERM	Phosphorylated ezrin-radixin-moesin proteins
PFA	Paraformaldehyde
RIPA	Radioimmunoprecipitation assay
ROCK	Rho/Rho-associated protein kinase
RPMI-1640	Roswell Park Memorial Institute medium 1640
siRNA	Small interfering RNA
SRC	SRC tyrosine kinase
TBST	Tris-buffered saline Tween 20
