# Supplementary material for: Analysis of the Role of the SRC Tyrosine Kinase and Podoplanin in the Process of Entosis
Source: Cancers (Basel). 2025 Sep 29;17(19):3173. doi: 10.3390/cancers17193173 (PMC12523579; doi:10.3390/cancers17193173)
Supplement: Supplementary file 1 [file cancers-17-03173-s001.zip › cancers-3876692-supplementary.pdf]

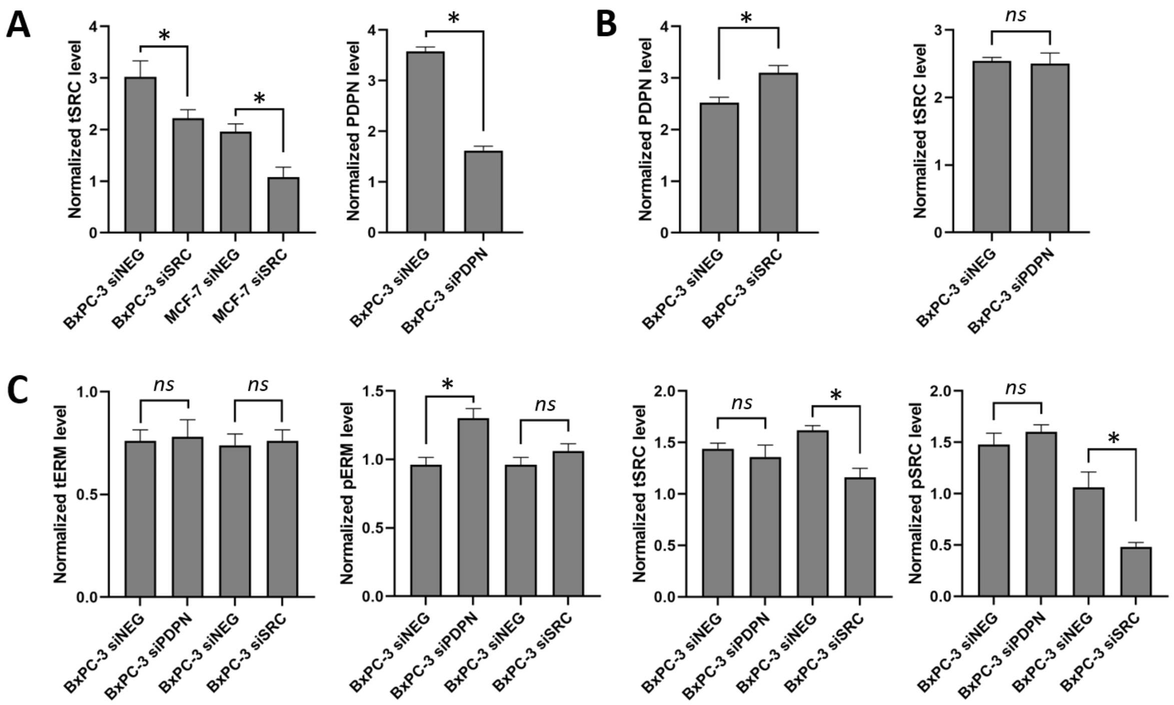

Figure S1. Densitometric analysis of Western blot results presented in the main manuscript:
(A) Yield of total SRC and PDPN in BxPC-3 and MCF-7 cells presented in Figure 1;
(B) Effect of *SRC* or *PDPN* depletion in BxPC-3 cells on the yield of total SRC or PDPN, respectively, presented in Figure 7;
(C) Analysis of the yield of phosphorylated and total ERM proteins and phosphorylated and total SRC after depletion of *PDPN* or *SRC* in BxPC-3 cells presented in Figure 8.
siNEG – control cells; siPDPN – cells with depleted *PDPN*; tSRC – total SRC; pSRC – phosphorylated SRC; tERM – total ERM (ezrin-radixin-moesin); pERM – phosphorylated ERM proteins.
*$p < 0.05$; *ns* – non-significant.
